# Knowledge and practice of nurses towards prevention of pressure ulcer and associated factors in Gondar University Hospital, Northwest Ethiopia

**DOI:** 10.1186/s12912-015-0076-8

**Published:** 2015-05-16

**Authors:** Nurhusien Nuru, Fisseha Zewdu, Senafikish Amsalu, Yohannes Mehretie

**Affiliations:** Emergency Department, Metema Hospital, Gondar, Ethiopia; Department of Nursing, University of Gondar, Gondar, Ethiopia; Department of Reproductive Health, Institute of Public health, University of Gondar, Gondar, Ethiopia; Department of Nursing, Wolaita Sodo University, Wolaita Sodo, Ethiopia

**Keywords:** Pressure ulcer, Knowledge and practice

## Abstract

**Background:**

Pressure ulcers are the common conditions among patients hospitalized in acute and chronic care facilities and impose significant burden on patients, their relatives and caregivers. Pressure ulcers have been described as one of the most costly and physically debilitating complications since the 20^th^ century. The pain and discomfort due to pressure ulcer prolongs illness, rehabilitation, time of discharge and even contribute to disability and death. This study was aimed to assess knowledge, practice and factors associated with pressure ulcer prevention among nurses in Gondar University Hospital, North-west Ethiopia.

**Method:**

An institution-based cross-sectional survey was conducted from March 15 - April 10, 2014 among 248 nurses in Gondar University hospital. A pretested and structured self-administered questionnaire was used for data collection. Data were entered using EPI info version 3.5.3 statistical software and analyzed using SPSS version 20 statistical package. Descriptive statistics was used to describe the study population in relation to relevant variables. Bivariate and multivariate logistic regression was also carried out to see the effect of each independent variable on the dependent variable.

**Result:**

Nearly half (54.4 %) of the nurses had good knowledge; similarly 48.4 % of them had good practice on prevention of pressure ulcer. Educational status [Adjusted Odds Ratio (AOR) = 2.4, 95 % CI (1.39-4.15)], work experience [AOR = 4.8, 95 % CI (1.31-10.62)] and having formal training [AOR = 4.1, 95 % CI (1.29-9.92)] were significantly associated with knowledge on prevention of pressure ulcer. While, satisfaction with nursing leadership [AOR = 1.9, 95 % CI (1.04-3.82)], staff shortage [AOR = 0.07, 95 % CI (0.03-0.13)] and inadequate facilities and equipment [AOR = 0.4, 95 % CI (0.19-0.83)] were found to be significantly associated with the practice on prevention of pressure ulcer.

**Conclusion:**

Knowledge and practice of the nurses regarding prevention of pressure ulcer was found to be inadequate. Having higher educational status, attending formal training and being experienced were positively associated with knowledge; while shortage of facilities and equipments, dissatisfaction with nursing leadership and inadequate staff number showed negative association with practice of nurse’s pressure ulcer prevention. In-service training and upgrading courses are some of the important steps to improve nurses’ knowledge and practice on prevention of ulcer pressure.

**Electronic supplementary material:**

The online version of this article (doi:10.1186/s12912-015-0076-8) contains supplementary material, which is available to authorized users.

## Background

Pressure ulcers are the common conditions among patients hospitalized in acute and chronic care facilities and impose a significant burden on patients, their relatives and caregivers [1]. Now days, pressure ulcers are recognized worldwide as one of the five most common causes of harm to patients and preventable patient safety problem. Also increasingly described as an indicator of the quality of care provided by health care organizations [2-4].

Pressure ulcers have been described as one of the most costly and physically debilitating complications since the 20^th^ century. The pain and discomfort of pressure ulcer delays rehabilitation, prolongs illness and timing of discharge, and also contribute to disability and death. These dramatically raise health care costs as a result of the need for supplies and nursing hours [5]. Moreover, health care budgets expend billion of dollars worldwide on prevention and treatment of patients with extended hospital stays from pressure ulcer development [6]. It has been estimated that the cost of treating pressure ulcer is 2.5 times higher than the cost of preventing [7]. In USA, pressure ulcers remain a major health problem affecting approximately 3 million adults [8]. A systematic review of 31 studies found that pressure ulcers significantly limit many aspects of an individual’s well-being, including general health and physical, social, financial and psychological quality of life [9]. So, the burden of pressure ulcers goes beyond increasing health care costs to loss of life [10].

According to an international literature, it has been identified that nurses’ knowledge of the prevention of pressure ulcers is poor, which is reflected in their practices as they do not comply with best practice guidelines [11]. Study conducted in Sweden on nurses’ knowledge and practice of existing guidelines on prevention of pressure ulcer found that, majority of them had inadequate knowledge and practice to implement guidelines [12]. Similarly, a study in Belgian Hospital found that knowledge of nurses about the prevention of pressure ulcers was inadequate [13]. Poor knowledge and practice of nurses have its own significant contribution for higher prevalence of pressure ulcers [14]. Moreover, a study in Bahir Dar, Ethiopia found that a total of 71 pressure ulcers were detected in 422 patients, with the prevalence rate of 16.8 %. The prevalence of pressure ulcer was higher in male respondents than in female respondents [15].Because, even if the prevention of pressure ulcers is a multidisciplinary responsibility, usually nurses play a major role and it is considered to be an essential part of nursing care in high income countries. Thus preventing ulcer should be the goal of all nurses [16] but it is rarely researched in low income countries like Ethiopia.

In Ethiopia there is lack of evidence on nurses’ knowledge and practice of pressure ulcer prevention. Therefore, this study set out to assess the level of nurses’ knowledge and practice on prevention of pressure ulcer and thereby generate appropriate information that can be used by program managers and stakeholders in the prevention and interventions of pressure ulcer.

## Methods

### Study design and set up

An institutional based cross sectional study was conducted among nurses working in Gondar University hospital. The hospital is located in Gondar town, Amhara regional state Northwest Ethiopia. Gondar is 740 km from the capital Addis Ababa. The hospital was established in 1954 and provides outpatient and inpatient services for more than 5 million peoples living in its catchment area.

### Sample size and sampling procedure

The sample size was determined by using single population proportion formula with the assumption of: 50 % proportion, 95 % confidence level and 5 % margin of error. Given that the source population was less than 10,000 correction formula was used and 5 % non-response was added, making the final sample size 201. Since the total number of nurses working in the hospital was 255, the study involved all of them to increase the power of the study.

### Data collection tool and procedure

Data were collected using a structured and pretested self administered questionnaire. The questionnaire and the consent form were prepared in English. Participants were asked 22 knowledge based and 22 practice based questions to assess their level of knowledge and practice towards prevention of pressure ulcer. Four midwife nurses and Public Health Officers collected the data with close supervision. Data quality was controlled by giving trainings and appropriate supervisions for data collectors. The overall supervision was carried out by the principal investigator. A pre-test was conducted using 6 % of the questionnaire on nurses who were working in Bahir Dar Referral Hospital. Appropriate modifications were made after analyzing the pretest result before the actual data collection.

The appropriateness of the instrument was measured through a pre-testing exercise, and the constraining factors were rectified. Prior to applying the survey instrument, the researchers engaged different expert reviewers as subject matter specialists at Gondar University Hospital to evaluate and finalize the instrument. Regarding to the reliability, the study used Cronbach’s coefficient alpha to measure consistency, complementarily and correlation coefficient. To generate the Cronbach’s alpha results, validation of the instrument was conducted through a pilot study and the results obtained had an overall Cronbach’s alpha of (r) = 0.76.

### Operational definitions

#### Pressure ulcer

a lesion of skin or underlying tissues by direct unrelieved pressure for more than 3 hours on the skin.

#### Good knowledge

Nurses, who scored above the mean score of the knowledge questions, were considered as having good knowledge on pressure ulcer prevention. But in the contrarily, those scored below the mean value considered as having poor knowledge towards prevention of pressure ulcer.

#### Good practice

Nurses who scored above the mean score of the practice questions related to prevention of pressure ulcer were considered to have good practice. But in the contrarily, those who scored below the mean score were considered as having poor practice towards prevention of pressure ulcer.

### Data processing and analysis

The questionnaires filled by the nurses were checked for completeness and entered into EPI INFO version 3.5.3 statistical software and then exported to SPSS version 20 for further analysis. Descriptive statistics was used to describe the study population in relation to relevant variables. Both bivariate and multivariate logistic regression models were used to identify associated factors. Odds Ratios and their 95 % Confidence Intervals were computed and variables with p - value less than 0.05 were considered as significantly associated with the outcome variable.

### Ethical considerations

Ethical clearance was obtained from University of Gondar, Ethical Review Committee of Department of Nursing. A formal letter of cooperation was written to Gondar University Hospital. After the purpose and objective of the study had informed, verbal consent was obtained from each study participant. Data were kept anonymously in the distributed questionnaire in order to keep confidentiality.

## Results

### Socio-demographic characteristics of the study participants

Out of the expected 255 respondents, 248 agreed to participate in the study, yielding a response rate of 97.3 %. The mean age of the respondents was 28.25 years (SD = 5.1). Around half (53.6 %) were single. Nearly half of them (50.8 %) were males, two third (62.5 %) of the nurses had bachelor degree. Most (92.7 %) had working experience of less than 10 years (Table 1).

**Table 1:** Percentage distribution of the study participants by of Socio Demographic Characteristics, Gondar University Hospital, Northwest Ethiopia, 2014

Variables	Frequency (n= 248)	Percentage (100%)	Mean with SD
Sex			
Male	126	50.8	
Female	122	49.2	
Age (in years)			
20-30	206	83.1	
31-40	31	12.5	28.25 ± 5.1
≥41	11	4.4	
Marital status			
Single	133	53.6	
Married	122	45.2	
Divorced	3	1.2	
Level of education			
Diploma in nursing	93	37.5	
Bachelor in nursing	155	62.5	
Service experience			
≤10	230	92.7	
11-20	18	7.3	4.33 ±3.5

### Organizational factors on prevention of pressure ulcer

Majority (91.1 %) of the nurses had not received any formal training and 223 (89.9 %) of them were not using any existing guidelines about risk assessment and prevention of pressure ulcer. More than half of the nurses (53.2 %) were not satisfied with the nursing leadership of the hospital. More than three quarter (78.6 %) of the nurses disagreed to the time given for each patient care. While Less than half (43.5 %) of the nurses reported staff shortage, big majority (88.3 %) of them agreed the hospital had inadequate facilities and equipments (Table 2).

**Table 2:** Percentage distribution of nurses’ organizational factors, Gondar University Hospital, Northwest Ethiopia, 2014

Variables	Frequency (n=248)	Percentage (%)
Formal training on pressure ulcer		
Yes	22	8.9
No	226	91.1
Guidelines on pressure ulcer		
Yes	25	10.1
No	223	89.9
Nursing leadership		
Satisfied	116	46.8
Not satisfied	132	53.2
Time for patients		
Agree	53	21.4
Disagree	195	78.6
Staff shortage		
Agree	108	43.5
Disagree	140	56.5
Inadequate facilities and equipments		
Agree	219	88.3
Disagree	29	11.7

### Knowledge of pressure ulcer prevention

**Fig. 1:**
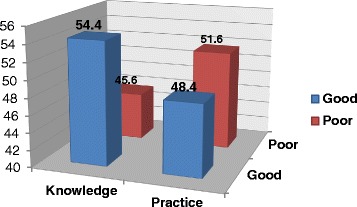
Nurses’ knowledge and practice regarding to prevention of pressure ulcer, Gondar University Hospital, Northwest Ethiopia, 2014

Participants were asked 22 questions to assess their knowledge on pressure ulcer prevention, and they were categorized in to two groups based on their score in relation to the mean. The mean score was 12.79 (SD = 3.21). More than half (54.4 %) of the respondents were found to have good knowledge, while a substantial proportion (45.6 %) of the respondents were not (Fig. 1).

From the six dimensions of knowledge regarding prevention of pressure ulcer, the nurses had a poor knowledge on three including risk assessment, skin care and management for mechanical loads. But, they possessed a good knowledge on factors related to pressure ulcer formation (M = 71.9, SD = 25.2), benefit of nutrition to maintain healthy skin (M = 69.9, SD = 29.1) and importance of staff training (M = 60.7, SD = 34.2) (Table 3).

**Table 3:** Frequency and percentage distribution of the nurses’ knowledge on prevention of pressure ulcer (N= 248) in Gondar University Hospital, Northwest Ethiopia, 2014

Nurses knowledge regarding to pressure ulcer	Rate of nurse’s knowledge			
	Correct		Incorrect	
	n	%	n	%
1. High loading pressure is the contributing factor for pressure ulcer formation	175	70.6	73	29.4
2. Immobility is the most important factor for pressure ulcer Formation in an 80- years old man with fracture hip and bedridden	158	63.7	90	36.3
3. Feces is the favorable environment for bacterial growth in the form of maceration for a young man having head injury with unconsciousness	184	74.2	64	25.8
4. Low albumin is the critical determinant for pressure ulcer formation	196	79.0	52	21.0
5. Head to toe skin assessment is an assessment procedure for a patient with spinal cord injury who is at high risk for pressure ulcer development	150	60.5	98	39.5
6. Braden scale is the risk assessment scale for pressure ulcer development	138	55.6	110	44.4
7. Risk assessment scale is an appropriate method for assessing an individual who is at risk for pressure ulcer development	125	50.4	123	49.6
8. Partial skin loss with blister and abrasion is correct answer for the sign of stage II pressure ulcer	199	80.2	49	19.8
9. Pale , red , or blue – gray discoloration on the skin is the sign for pressure ulcer development	97	39.1	151	60.9
10. Topical cream is appropriate method for skin care	40	16.1	208	83.9
11. Turn position for every 2 hours is significant activity for protecting skin damage	194	78.2	54	21.8
12. Cleansing soil and using skin barrier cream or lotion activity is appropriate for preventing maceration for a 78 – years old man having a stroke with hemiplegic	147	59.3	101	40.7
13. Lift up the patient without dragging is a correct practice for maintaining skin integrity	96	38.7	152	61.3
14. Use pillow under the patients leg to prevent heel ulcer	69	27.8	179	72.2
15. Vitamin C and E is important to maintain healthy skin	194	78.2	54	21.8
16. High protein and high calorie needs to be offered to a 85- years Old bedridden patient who has BMI < 18.5	167	67.3	81	32.7
17. Serum albumin is an appropriate lab test for nutritional assessment of pressure ulcer patient	159	64.1	89	35.9
18. Turn position is an appropriate nursing care for managing mechanical load	159	64.1	89	35.9
19. Lift patient without dragging is appropriate activity to reduce friction for an 80- years old man having fracture hip with skeletal traction	160	64.5	88	35.5
20. Elevate the head of bed < 30^0^ is the activity for reducing shearing force	66	26.6	182	73.4
21. Schedule of Turing position is necessary educational Information for reducing pressure ulcer formation	168	67.7	80	32.3
22. In- service training on pressure ulcer prevention is the best Educational activity that enhances competency of staff nurses in preventing pressure ulcer	133	53.6	115	46.4

### Nurses’ practice regarding to prevention of pressure ulcer

By using 22 practice based questions, the mean practice score of the respondents was found to be 12.16 (SD = 9.75) (Table 4). Nearly half (48.4 %) of the respondents had good practice; whereas the remaining 51.6 % respondents had poor practice of pressure ulcer prevention (Fig. 1).

**Table 4:** Frequency and percentage distribution of nurses’ practice on prevention of pressure ulcer (N=248) in Gondar University Hospital, Northwest, 2014

Nurses practice on pressure ulcer prevention	Rate of nurse’s practice		
	Always (%)	Sometimes (%)	Never (%)
1. I Observe how other nurses assess the risk factors	4.8	42.3	52.9
2. I identify common contributing factors	8.1	41.1	50.8
3. I do a skin assessment	5.2	18.1	76.7
4. I use risk assessment scale	5.6	14.9	79.5
5. I document all data	12.9	17.7	69.4
6. I assess and provide management of pain	41.1	30.6	28.3
7. I perform skin care as a routine work	15.7	44.4	39.9
8. I place the pillow under the patient’s leg	7.3	32.7	60.0
9. I use water filled glove under the patient’s leg	6.5	31.0	62.5
10. I use or advice caregiver to use creams or oils	4.4	28.6	67.0
11. I pay more attention to pressure points	17.7	32.3	50.0
12. I perform lab tests	5.2	13.3	81.5
13. I provide vitamin and food	8.9	30.2	60.9
14. I monitor a protein and calorie diet	4.0	27.4	68.6
15. I avoid dragging	39.5	23.8	36.7
16. I always use a special mattress	10.9	21.0	68.1
17. I avoid massage	42.7	18.1	39.2
18. I avoid using donut – shape (ring) cushion	34.7	23.8	41.5
19. I turn a patient position every two hours.	12.9	31.9	55.2
20. I put pillows under the patient’s leg ankle	8.1	27.8	59.1
21. I always attend seminars	4.4	15.7	79.9
22. Give advice to the patient or caregiver	8.9	30.6	60.5

### Factors associated with nurses’ knowledge regarding to prevention of pressure ulcer

Level of education, length of work experience and formal training on prevention of pressure ulcer were found to have significant and independent effect on nurses’ knowledge regarding to prevention of pressure ulcer, while availability of guidelines about pressure ulcer prevention was not significantly associated at p-value of < = 0.05.

Those nurses who had bachelor degree were 2.4 times [AOR = 2.4, 95 % CI (1.39-4.15)] more likely to have a good knowledge on prevention of pressure ulcer as compared to those nurses who had diploma. Nurses who had work experience of 11-20 years were 4.8 times [AOR = 4.8, 95 % CI (1.31-10.62)] more likely to have good knowledge than nurses with less than 10 years of work experience.

Those nurses who had formal training about pressure ulcer were 4.1 times [AOR = 4.1, 95 % CI (1.29-9.92)] more likely to have good knowledge than those nurses who did not took training about pressure ulcer (Table 5).

**Table 5:** Bivariate and Multivariate analysis of factors associated with nurses’ knowledge regarding to prevention of pressure ulcer, Gondar university Hospital, Northwest Ethiopia, 2014

	Knowledge			
	Good, n (%)	Poor, n (%)	COR (95% CI)	AOR (95% CI)
Level of education				
Diploma	39(41.9)	54(58.1)	1.00	1.00
Bachelor	96(61.9)	59(38.1)	2.3(1.33-3.81)	2.4(1.39-4.15)
Work experience				
1-10	120(52.2)	110(47.8)	1.00	1.00
11-20	15(83.3)	3(16.7)	4.6(1.29-10.26)	4.8(1.31-10.62)
Training				
Yes	18(81.8)	4(18.2)	4.2(1.38-9.78)	4.1(1.29-9.92)
No	117(51.8)	109(48.2)	1.00	1.00
Guidelines				
Yes	20(80.0)	5(20.0)	3.8(1.36-10.36)	
No	115(51.6)	108(48.4)	1.00	*

*Not significant in the multivariate analysis (back ward stepwise logistic regression)

### Factors associated with nurses’ practice of pressure ulcer prevention

Satisfaction with nursing leadership, Having formal training on pressure ulcer prevention, staff shortage and inadequate facilities and equipments were found to have significant and independent effect on practice of nurses’ towards prevention of pressure ulcer, while level of education, length of work experience were not significantly associated at p-value of < = 0.05.

Nurses who were satisfied with nursing leadership were around 2 times [AOR = 1.9, 95 % CI (1.04-3.82)] more likely to have good practice of pressure ulcer prevention when compared to those who were not. Study participants who agreed about staff shortage in the work place were 93 % [AOR = 0.07, 95 % CI (0.03-0.13)] less likely to have good practice than nurses who disagreed about the shortage of staff. Moreover, respondents who agreed about inadequate facilities and equipments in the work place were 60 % [AOR = 0.4, 95 % CI (0.19-0.83)] less likely to have good practice towards prevention of pressure ulcer as compared to those who were disagree about inadequate facilities and equipments (Table 6).

**Table 6:** Bivariate and Multivariate analysis of factors associated with nurses’ practice regarding to prevention of pressure ulcer, Gondar University Hospital, Northwest Ethiopia, 2014

Variables	Practice			
	Good, n	Poor, n	COR (95% CI)	AOR (95% CI)
Level of education				
Diploma	43	50	1.00	
Bachelor	77	78	1.2(1.01-2.92)	*
Work experience				
1-10	109	121	1.00	
11-20	11	7	1.7(1.34-4.66)	*
Time allocation				
Agree	26	27	1.00	
Disagree	94	101	0.9(0.53-1.77)	*
Nursing leadership				
Satisfied	73	43	3.1(1.83-5.16)	1.9(1.04-3.82)
Not satisfied	47	85	1.00	1.00
Staff shortage				
Agree	16	92	0.06(0.03-0.12)	0.07(0.03-0.13)
Disagree	104	36	1.00	1.00
Inadequate facilities				
Agree	99	120	0.3(0.13-0.74)	0.4(0.19-0.83)
Disagree	21	8	1.00	1.00

*Not significant in the multivariate analysis (back ward stepwise logistic regression)

## Discussion

Prevention of pressure ulcers is an indicator of quality of care. Nursing care has a major effect on pressure ulcer development and prevention. Hence, Pressure ulcers are a major nurse-sensitive outcome [16]. So this study was aimed to describe the level of nurse’s knowledge and practice on prevention of pressure ulcers and its associated factors in Gondar University Hospital, Northwest Ethiopia.

In this study, 54.4 % of the participants were found to be knowledgeable. While substantial proportions 45.6 % were not, this is inadequate. Because, as they are nurses working in recognized teaching referral hospital, and are expected to be well experienced, this level of knowledge is below the anticipated. The finding of this study is comparable with other studies conducted in different parts of the world. In a study conducted in Turkey the mean score of correct answer was 48.85 % [17] and the study conducted in Belgian hospital revealed that the mean knowledge score was 49.7 % [13]. Similarly, the study conducted in Bangladesh indicated that the overall nurses’ knowledge on pressure ulcer prevention were found to be 57.79 % [18] and the other study conducted in one of the largest health insurance hospital in Alexandria, found that, the overall mean percentage score for nurses were below the minimum acceptable level [19].

Respondent’s formal education and training experience may be a factor related to this poor level of knowledge. Pressure ulcer prevention related content included in their curriculums might not be sufficient. In addition, lack of learning resources for nurses to update their knowledge would be another reason for the poor level of knowledge. Specifically in Ethiopia, there is a limited learning resource for nurses to update their knowledge. Moreover nursing journals are not available even at the nursing institutes or hospitals. Specific to this study 91.1 % of the participants had not received any formal training and 89.9 % of the nurses were not using any existing guidelines on risk assessment and prevention of pressure ulcer.

Respondent’s level of education was found to be significantly associated with knowledge of pressure ulcer prevention. This finding is in line with the study conducted in Sweden among registered nurses and licensed practicum nurses in which the registered nurses’ score were significantly higher than those of the licensed practicum nurses [20]. This could be attributed to the possibility that more educated respondents have a higher opportunity of exposure to different courses directly or indirectly related to prevention of pressure ulcer.

Respondents with work experience of 11-20 years had good knowledge when compared to those with work experience of ≤ 10 years. Similar finding was reported in study conducted in Nigeria; where years of experience were significantly associated with clinical practice and knowledge [21]. Other study done in Spain on Nurses’ knowledge and clinical practice of pressure ulcer care revealed that, the greater the working experience the higher the knowledge gained [22]. The reason might be nurses with more years of working experience have more chance to work with different professionals so that they can learn from their coworker’s experience. Also since they have more prolonged exposure to patient care, they have greater chance to learn how to prevent pressure ulcer even from their own mistakes as compared to those who have less years of working experience.

Nurses who took formal training on pressure ulcer prevention were found to have good knowledge than those who had not. Similarly in a study conducted in Swedish healthcare to assess knowledge, attitude and practice of nursing staff on pressure ulcer prevention; nurses who had training were more knowledgeable than those who did not [23]. This might be due to the fact that training increases the chance of the trainees to get up to date information about pressure ulcer related preventions.

Staff shortage is one of the factors associated to nurse’s practice in prevention of pressure ulcer. This study also favored the above claim in which, respondent’s practice of pressure ulcer prevention was found to be poor which was less than 50 %. Similarly study conducted in England showed that, majority of the nurses reported lack of staff and time as barrier to implement effective care practices related to prevention of pressure ulcer [24]. The poor practice can be explained by the fact that, shortage of nursing staff limits the working time available for each patient’s care. Especially in countries like Ethiopia where number of health professionals is near to the ground, inadequate nurse to patient ratio may limit the implementation of quality care related to pressure ulcer prevention.

In this study more than three fourth of nurses did not use a risk assessment scale. Similarly the study conducted in Sydney found that 79 % of the nurses did not use any assessment tool to identify patients with at risk of pressure ulcer [25]. This can be explained by lack of evidence based nursing practice and in-service training on prevention of pressure ulcer.

Respondents who were satisfied with the nursing leadership had good practice as compared to those who were not. Possible reason for this result might be nurses who are satisfied with the nursing leadership are happier on their working environment, so that they are motivated to invest all their knowledge and experiences on practices related to prevention of pressure ulcer.

Inadequate facilities and equipments in the workplace were associated with poor practice on prevention of pressure ulcer. This might be due to the fact that limited access to adequate facilities and equipments may hinder nurse’s motivation and ability to prevent patients from developing pressure ulcer.

Using a self reported questionnaire to examine the nurses’ practice towards prevention of pressure ulcer was the main limitation of this study.

## Conclusions

Nurses’ knowledge and practice regarding prevention of pressure ulcer was found to be inadequate. Having higher educational status, attending formal training, being more experienced showed a positive and significant association with knowledge; whereas inadequate facilities and equipments, dissatisfaction with the nursing leadership and staff shortage and were found to be associated with poor practice of pressure ulcer prevention. In-service training, upgrading courses and ensuring availability of the necessary facilities and equipments are some of the important steps to improve nurses’ knowledge and practice regarding to prevention of pressure ulcer.
